# Peer Volunteers’ Journeys Through Training and Engagement in Older Adult Communities: Descriptive Qualitative Study

**DOI:** 10.2196/71810

**Published:** 2025-06-13

**Authors:** Shefaly Shorey, Jeremy Soon Leong Seow, Rathi Mahendran, Geok Hua Wee, Lee Gan Goh, Ee Heok Kua

**Affiliations:** 1 Alice Lee Centre for Nursing Studies, Yong Loo Lin School of Medicine, National University of Singapore, 10 Medical Dr, Singapore, 117597, Singapore, 65 65163320; 2 Department of Psychological Medicine, Yong Loo Lin School of Medicine, National University of Singapore, Singapore, Singapore; 3 Principle Mindful Awareness Trainer (MAP), Age-Well Everyday Programme (AWE), Singapore, Singapore; 4 Division of Family Medicine, Yong Loo Lin School of Medicine, National University of Singapore, Singapore, Singapore

**Keywords:** psychological first aid, PFA training, peer volunteers, mental health, psychosocial support, older adults

## Abstract

**Background:**

The rising prevalence of mental health conditions such as depression and anxiety among the aging population underscores the need for accessible and effective psychosocial support, particularly for community-dwelling older adults who face barriers like social stigma and limited mental health literacy. Peer volunteers have emerged as a promising resource to support these individuals; yet, they often lack the requisite training for effective intervention.

**Objective:**

This study aims to explore the experiences of peer volunteers who participated in a Psychological First Aid training program.

**Methods:**

Using a descriptive qualitative research design, semistructured interviews were conducted with 13 older adults between September and October 2024, and data were thematically analyzed.

**Results:**

Three themes were identified: (1) dimensions of volunteerism from motivations to resistance, (2) empowerment through collaborative learning, and (3) recommendations for designing inclusive, holistic training programs.

**Conclusions:**

The findings of this study showed positive outcomes such as personal growth and strengthened social connections among participants. However, enhancements in teaching methods, logistical arrangements, and session regularity are recommended to optimize the Psychological First Aid program. These insights can guide the development of more robust training models to support both peer volunteers and the older adult communities they serve.

## Introduction

The global population of older adults has grown significantly in recent years. In Singapore, the proportion of citizens aged 65 years and older increased to 19.1% in 2023 and is projected to reach 24.1% by 2030 [1]. Globally, approximately 14% of older adults live with mental health conditions, with depression and anxiety being the most common [2]. This trend is mirrored in Singapore, where issues such as depression, dementia, and subsyndromal depression are increasingly prevalent among older adults [3]. Social isolation, multimorbidity, and low socioeconomic status have been identified as key contributors to the development of mental health conditions in older adults. These factors are associated with severe consequences, including increased suicide risk, diminished quality of life, and heightened susceptibility to cognitive disorders such as dementia [4-7]. Unfortunately, mental health conditions in this population often remain undiagnosed or underreported due to stigma and limited mental health literacy, leading to delays in treatment and intervention [8,9].

Volunteerism has been recognized as an effective approach to improving the mental well-being of older adults. Programs such as AmeriCorps Seniors and Age UK internationally, and SG Cares and Lions Befrienders locally, provide platforms for older adults to engage socially and meaningfully, helping to reduce social isolation and promote active aging [10-15]. Despite these benefits, studies conducted in Portugal and the United Kingdom reveal that volunteers often face challenges due to insufficient training to support individuals with mental health conditions [16,17]. Structured training programs have been increasingly recognized for their ability to prepare peer volunteers to provide effective psychosocial support. Peer volunteers’ training has been widely used in various settings to help individuals cope with potentially traumatic events, reduce distress, and foster a sense of safety while supporting others [18,19]. Such programs have also proven beneficial not only in humanitarian aid situations but also in enhancing volunteers’ ability to offer timely, empathetic, and informal support [20,21]. Formal training equips volunteers with the knowledge and skills necessary to provide effective support to individuals in distress while also addressing the volunteers’ own needs [22].

However, most studies examining peer volunteer training have been conducted in Western contexts, highlighting a critical gap in understanding its application and effectiveness in Asian settings [23-25]. Given that Asian cultures are deeply rooted in collectivism and family-centered caregiving, there is strong evidence that Western models shaped by individualism and structured volunteer systems may not fully translate to these contexts [26]. Considering the growing prevalence of mental health conditions among Singapore’s older adults and the potential benefits of formal training programs, there is an urgent need to implement and evaluate such initiatives locally.

This study aims to explore the experiences of peer volunteers who participated in a Psychological First Aid (PFA) training program designed to support community-dwelling older adults in Singapore. Specifically, it addresses the research question: “What are the experiences of peer volunteers who completed the PFA programme?” The findings will inform the refinement of culturally relevant and effective training strategies to enhance psychosocial support for both volunteers and the older adult population in Singapore.

## Methods

### Study Design

A descriptive qualitative research design was used to explore the experiences of peer volunteers who participated in a PFA program. The use of a qualitative design is prudent as it allows participants to share “thick” or “rich” descriptions, providing deeper insights into subjective topics [27]. The Consolidated Criteria for Reporting Qualitative Research (COREQ) checklist guided the reporting of this qualitative study [28] (Table S1 in Multimedia Appendix 1).

### Sampling Strategy and Eligibility

Convenience sampling was used to recruit eligible participants who met the following criteria: (1) older adults aged 50 to 95 years, (2) participated in the PFA program, and (3) spoke and read English.

### PFA Program

The PFA program was developed based on foundational literature [29-31] and insights from a multidisciplinary research team. This team comprised psychiatrists, psychologists, nurses, and peer volunteers with extensive experience supporting community-dwelling older adults. Their prior primary and secondary research studies formed the basis of the PFA intervention design.

The program consisted of 4 half-day workshops, each lasting 4 hours, and conducted face-to-face over consecutive weekends. The workshops were held in a conducive community space and addressed a range of topics, including common mental health disorders in Singapore, psychosocial needs of older adults and peer volunteers, skills in empathetic listening, low-intensity psychological support, mindfulness training, identifying individuals at risk, and strategies for facilitating connections to professional care.

Workshops were delivered by a diverse team of trainers, including psychiatrists, nurses, experienced peer volunteers, and mindfulness instructors. Peer volunteers involved in earlier training sessions also contributed as facilitators, adding a practical perspective to the training content. The primary objective of the PFA program was to enable early detection of mental health issues among community-dwelling older adults. By equipping volunteers with these skills, the program also aimed to enhance the resilience and coping strategies of both the older adults and the volunteers themselves.

### Ethical Considerations

The study received ethical approval from the institutional review board of the National University of Singapore (NUS-IRB-2024‐506), which serves as the ethics board for the participating institution. Prior to recruitment, participants were provided with detailed information sheets outlining the study’s objectives and their roles. Written informed consent was obtained, and voluntary participation was emphasized. To ensure anonymity and confidentiality, each participant’s data were coded using unique serial numbers ranging from 1 to 13.

### Data Collection

Data collection for the study was conducted between September and October 2024. All 20 peer volunteers who participated in the first batch of the PFA program were invited to join the study. Invitations were extended by a study administrator independent of the research team. Volunteers who expressed interest were introduced to the research team. A male research assistant (independent of the PFA program and participants) explained the study details to the interested participants, provided the participant information sheet, and obtained written informed consent. Subsequently, one-on-one interviews were arranged. Participants could choose between web-based or in-person interviews based on their preference. Before the interview, sociodemographic data such as age, gender, citizenship, ethnicity, religion, marital status, number of children, education level, employment status, and volunteering duration were collected.

A male research assistant (JS) with a BSc (Hons) in Nursing and no dependent relationship with the participants, developed the semistructured interview guide guided by literature review [32,33] and conducted the interviews to ensure consistency. Additionally, the same research assistant recorded field notes during each session. The guide included open-ended questions focusing on participants’ experiences with the PFA program and their volunteerism journey (eg, “Would you like to share any personal experiences or stories that inspired you to join this PFA programme?” and “How was your experience with the PFA programme?”). All interviews were conducted via Zoom (Zoom Video Communications), and consent was obtained from participants for audio and video recording. Transcripts were transcribed verbatim from the recordings. To ensure rigor, the research assistant (JS) received qualitative research training under the guidance of a PhD-trained researcher (SS) with extensive qualitative experience. A pilot interview was conducted with one peer volunteer to evaluate the interview guide’s flow and relevance, aligning it with the research objectives; no modifications were needed, and the data from this pilot interview was included in the analysis as it contributed valuable insights to the study. A total of 13 interviews were conducted with no attrition, with durations ranging from 36 to 101 minutes (mean of 59.5 min). Data saturation was reached after the 11th interview, as no new concepts emerged [34]. Two additional interviews were conducted to confirm saturation. The interview guide is provided in Table S2 in Multimedia Appendix 1.

### Data Analysis

The data were analyzed using Braun and Clarke’s [35] 6-step framework for inductive thematic analysis. The process began with the verbatim transcripts being imported into Microsoft Word for organization and management. Two researchers (JS and SS) independently familiarized themselves with the data by reading and rereading the transcripts to gain a thorough understanding. Initial codes were generated by identifying meaningful units of information and relevant concepts within the data. These codes were systematically applied across all transcripts. Through an iterative process, the researchers grouped the codes into broader categories to develop initial themes and subthemes. These themes reflected the key patterns and narratives emerging from the data (Table S3 in Multimedia Appendix 1). The coding and thematic development were continuously refined through team discussions to ensure accuracy and depth. Any discrepancies in interpretation were resolved through consensus, ensuring a comprehensive and cohesive representation of the findings.

### Rigor

To ensure the rigor of this study’s findings, credibility, transferability, dependability, and confirmability were prioritized, as outlined by Cypress [36]. Credibility was maintained through prolonged engagement with participants during data collection and investigator triangulation during data analysis. This allowed for multiple perspectives to be considered and verified against the data. Transferability was established by providing thick, rich descriptions of participants’ experiences with the PFA program, supported by direct quotes. This approach enables readers to evaluate the applicability of the findings to other contexts. Dependability was reinforced by creating an audit trail, which included comprehensive documentation of interview transcripts, coding, and the analytical process. This ensures that the study can be independently reviewed and verified. Confirmability was achieved using a reflexive journal throughout the research process. Reflexivity, as highlighted by Teh and Lek [37], is a critical aspect of qualitative research that minimizes the influence of unconscious researcher biases during data interpretation. By maintaining this journal, the researchers ensured their reflections and decisions were transparent, bolstering the trustworthiness of the study [38].

## Results

### Overview

A total of 13 community-dwelling older adults participated in this study, and their sociodemographic characteristics are presented in Table 1. The mean age of the participants was 61.2 (SD 6.9) years. Most participants were female (9/13, 69%) and married (9/13, 69%), with all of them were Singaporeans (13/13, 100%). The group was predominantly composed of Malay (6/13, 46%) and Chinese (5/13, 38%) ethnic backgrounds. The participants’ employment status varied, with being retired (4/13, 31%), employed full-time (4/13, 31%), employed part-time (2/13, 15%), unemployed (2/13, 15%), and self-employed (1/13, 8%). The group had a balanced distribution of volunteerism experience, with 54% (7/13) having up to 1 year of experience, and 46% (6/13) having more than 1 year of experience.

**Figure 1.**
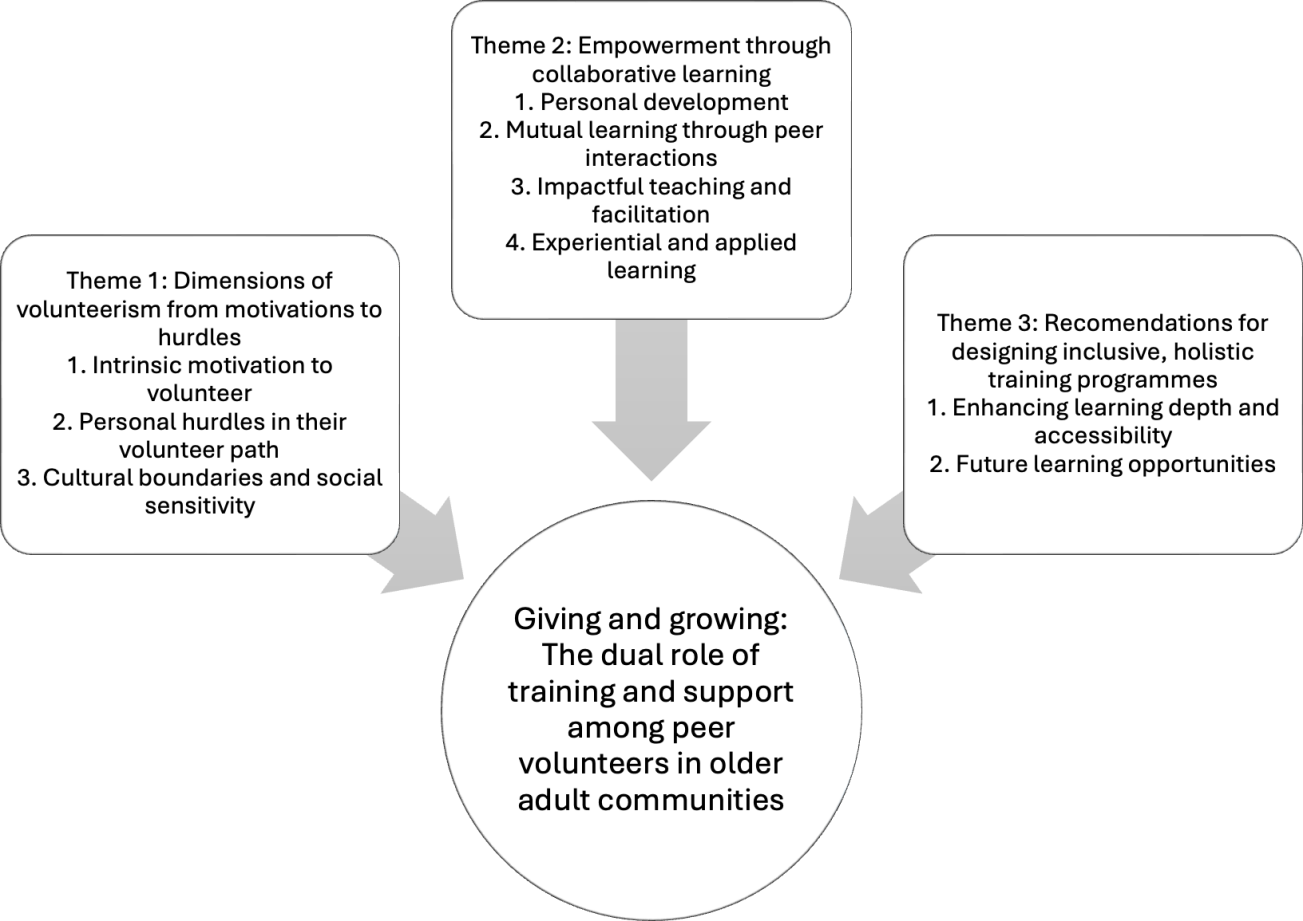
Older adults’ experience on the Psychological First Aid program and personal volunteering experience.

Three themes were identified from the data analysis, highlighting older adults’ experiences of the PFA program and the value it added to their volunteerism journey: (1) dimensions of volunteerism from motivations to hurdles, (2) empowerment through collaborative learning, and (3) recommendations for designing inclusive, holistic training programs. These themes were further supported by 9 subthemes (Figure 1).

**Table 1.** Participants’ sociodemographic characteristics (n=13).

Sociodemographic variables	Values
Age (years), mean (SD)	61.2 (6.9)
Sex, n (%)	
Female	9 (69)
Male	4 (31)
Nationality, n (%)	
Singaporean	13 (100)
Ethnicity, n (%)	
Chinese	5 (38)
Malay	6 (46)
Indian	1 (8)
Javanese	1 (8)
Marital status, n (%)	
Married	9 (69)
Single	3 (23)
Divorced	1 (8)
Length of marriage (years), n (%)	
0‐10	4 (31)
11‐20	1 (8)
21‐30	2 (15)
31‐40	3 (23)
>41	3 (23)
Highest education level, n (%)	
Secondary school	6 (46)
Polytechnic	2 (15)
University	5 (38)
Employment status, n (%)	
Employed full-time	4 (31)
Employed part-time	2 (15)
Self-employed	1 (8)
Unemployed	2 (15)
Retired	4 (31)
Volunteered as a peer volunteer (years), n (%)	
Up to 1	7 (54)
More than 1	6 (46)

### Theme 1: Dimensions of Volunteerism From Motivations to Hurdles

#### Overview

This theme highlighted older adults’ experiences from motivations to hurdles, which shaped both their initial decision to volunteer and their continued commitment. These were categorized into 3 subthemes: intrinsic motivation to volunteer, personal hurdles in their volunteer path, and cultural boundaries and social sensitivity.

#### Intrinsic Motivation to Volunteer

Most older adults shared their personal motivations for volunteerism. As the majority of older adults were either employed part-time or retirees, they had free time available. Hence, they expressed a strong desire to actively engage with the community to enhance their personal well-being by giving back to society and fostering friendships. Many older adults also shared the perceived benefits of volunteerism, from keeping their minds active and serving as a protection to prevent the onset of dementia.

*I volunteer as It’s to help myself to prevent having dementia because I'm learning something new...*

[K3, Female peer volunteer]

*I volunteer as It’s to help myself to prevent having dementia because I'm learning something new...*

[K3, Female peer volunteer]

Some older adults attributed their volunteerism motivations to the cultural values instilled by their families. They were raised by their parents to be benevolent and gracious, which fostered a strong desire to give back to society. As a result, the concept of volunteerism became deeply embedded in their upbringing.

*I see my father as a figure who do all this volunteer work in his life.… so it’s already in my blood*

[K5, Female peer volunteer]

Older adults attributed their decision to join the PFA program to the influence of their peers, highlighting the positive impact of fostering a sense of purpose and building meaningful friendships. For instance, one participant, who became a single mother during her teenage years, recounted the emotional struggles she faced, such as limited support from her family and peers, which negatively affected her emotional health. Having developed emotional resilience through overcoming these challenges, she felt inspired to volunteer and provide emotional support to others navigating similar difficulties.

*To help people because I had gone through a period of time [her teenage years] and from that time, it makes me want to touch more and especially on single moms*

[K13, Female peer volunteer]

Older adults shared that establishing meaningful engagement with their community residents was a significant challenge. Many encountered resistance when attempting to approach and offer support. Observing the reluctance of fellow older adults to seek help underscored for them the importance of acquiring proper training. They recognized that such training was crucial to effectively support and connect with others in their communities.

*I think it’s the resistance of the people like when we try to talk or whatever, there is certain resistance… that led me to learn more from this programme*

[K11, Female peer volunteer]

#### Personal Hurdles in Their Volunteer Path

Older adults highlighted the struggle of physical limitations, such as the lack of strength, despite being motivated to attend training courses to support others. This is an important consideration, as most community residents are older adults who may need assistance moving from one place to another. One participant shared that, due to her age and physical build, she is not as strong as she was in her youth and therefore needs to be more cautious when interacting with the older community residents or attending any courses.

*I don’t have much strength, things are very physical. I would not be able to help because … we may need to provide them physical support, like to support them so that they don’t fall down...maybe emotional support would do better [for me]…*

[K3, Female peer volunteer]

In addition, one participant shared that he was diagnosed with aphasia. This gravely impacted his communication abilities with both his peers and community residents. As a result, he struggled to express his needs efficiently.

*Sometimes my train of thoughts go out... sometimes when people talk to me, I can’t interpret properly… that sometime dampen me from opening up…*

[K10, Male peer volunteer]

#### Cultural Boundaries and Social Sensitivity

Many older adults identified cultural perceptions as a significant barrier in their volunteerism experiences. They faced cultural taboos, such as Muslims’ discomfort with certain terms used around older adults, including “forgetful” and concerns about interacting too much with the opposite gender. As a result, the volunteers, especially those of different ethnicities, had to be mindful of their behavior when approaching the community-dwelling older adults. Additionally, some volunteers highlighted that Muslim residents in the community often exhibit caution towards “outsiders” and show a preference for staying within their own social and ethnic circles, which limits their willingness to engage with others. These cultural dynamics present challenges in fostering broader social connections and offering support.

*For the Malay elderly how we want to get them, how are we going to approach them to ask them that, we can really give them help. That’s our challenge now.*

[K13, Female peer volunteer]

Gender dynamics were frequently discussed by older adults, who reported that supporting members of the opposite gender was often challenging and required careful consideration, particularly regarding body language and posture. Many volunteers found it easier to assist individuals of the same gender, such as a female peer volunteer supporting a female older adult, as they could more easily interpret nonverbal cues like body language and gestures. One female peer volunteer shared her discomfort when a male community resident exhibited inappropriate behavior, such as touching without consent. These gender-related challenges highlight the complexities involved in volunteer interactions and the need for sensitive handling of such situations in community support roles.

*Not easy to understand a male, what he’s thinking. A female, when you look into her eyes, you talk to her, you can understand more… not easy with males….*

[K1, Female peer volunteer]

Many felt the need for a chaperone, especially when interacting with the opposite gender, as a safety measure and to prevent unnecessary conflict. In one instance, one female peer volunteer experienced negative experiences from a wife due to her close rapport with the community resident.

*There is a gender difference [in our local setting] that we need to be more mindful. For female, we can touch the female, can consult but for male, we need to be more careful, more mindful on our gesture.*

[K11, Female peer volunteer]

### Theme 2: Empowerment Through Collaborative Learning

#### Overview

This theme highlighted elements that contributed to the empowerment of older adults through collaborative learning during the PFA program. These were supported by 4 subthemes as follows: personal development, mutual learning through peer interactions, impactful teaching and facilitation, and experiential and applied learning.

#### Personal Development

Older adults highlighted the benefits of participating in the PFA program. Many expressed how the knowledge acquired through the PFA program helped them in real-life situations, enabling them to better showcase their skills as peer volunteers. Topics such as grief and mindfulness were essential in their role as peer volunteers. This, in turn, fostered a sense of empowerment and personal growth among the older adults.

*It Was Nice, It Was Very Knowledgeable. I Must Say We Learn a Lot*

[K1, Female Peer Volunteer]

Additionally, the older adults reported maintaining a positive attitude toward learning. They expressed that learning is a continuous journey, and the knowledge they gained expanded their viewpoints.

*The… workshop where it really broadened our mind and then we learn more about things [eg how to support others better].*

[K2, Male peer volunteer]

#### Mutual Learning Through Peer Interactions

Older adults emphasized that the PFA program highlighted the importance of maintaining open and effective communication among peers. Through interactions during class and breaks, they had the opportunity to share personal experiences, which many participants found invaluable. This peer exchange allowed them to learn from each other’s experiences and mentally prepare for similar real-life situations in the future. The older adults also expressed that the learning process was reciprocal, with everyone eager to contribute and learn from one another.

*You get different ideas from them [peers attending PFA programme]. You learn more things from them, they learn from you.*

[K1, Male peer volunteer]

Additionally, some older adults shared that the class, which included individuals from various ethnic backgrounds such as Chinese, Malay, and Indian, provided a valuable opportunity to engage with and learn about different cultural practices. This allowed them to conceptualize and compare the differences or similarities between the cultures.

*The other group are Malays so then cultural wise, it’s quite different. I think to understand each other’s cultural difference… that was helpful*

[K3, Female peer volunteer]

#### Impactful Teaching and Facilitation

Dedicated facilitators consistently worked to build rapport with the older adults, helping to ease initial discomfort and encouraging open communication. Older adults highlighted that the facilitators were highly knowledgeable in their respective fields. They were committed to sharing their knowledge on various topics such as handling difficult situations and mindfulness, which made the older adults feel comfortable asking questions whenever they had any doubts. With that, this fostered communication and the older adults were able to maximize the knowledge gained throughout this PFA program.

*Think the way they [facilitators] present and they deliver the topic well, yeah and they are very knowledgeable on the topic… you feel motivated.*

[K11, Female peer volunteer]

Additionally, many older adults highlighted that they were inspired by the facilitators’ presentation style. The facilitators’ consistent engagement, well-paced sessions, body language, tone, and delivery made the learning process more accessible and understandable for older learners. The older adults also valued the facilitators’ sharing of personal experiences, as it allowed them to learn from real-life situations.

*I learn from it and [blinded] because she’s a mindfulness teacher and I’m learning to teach mindfulness. So of course I also want to learn all the right things from her.*

[K3, Female peer volunteer]

#### Experiential and Applied Learning

Many older adults favored experiential learning over traditional lecture-style delivery. During the PFA program, they were able to actively participate in hands-on activities such as role-plays and this helped them to better visualize and retain the concepts taught.

*The role play, it showed you like reality what could happen or what will happen or what can happen. That was really like an experience, very good because learning from slides and all is quite normal.*

[K1, Female peer volunteer]

Additionally, participants reported that the role-playing exercises played a crucial role in boosting their confidence in handling real-life situations. By simulating real-world scenarios, they were able to “experience” these situations in a controlled environment. This allowed them to observe nonverbal cues and body language, which heightened their awareness of their own nonverbal communication when offering support. The interactive nature of the role-playing activities also facilitated communication among the older adults, helping to break the ice and foster connections.

*When you have a role play, you really understand it more. You sometimes put yourself into that shoes and see how it is like.*

[K1, Female peer volunteer]

### Theme 3: Recommendations for Designing Inclusive, Holistic Training Programs

#### Overview

This theme highlighted recommendations to enhance the PFA program by addressing the varied needs, backgrounds, abilities, and learning styles of the participants. This theme is supported by 2 subthemes as follows: depth and quality of learning, and future learning opportunities.

#### Depth and Quality of Learning

A few older adults expressed that the content covered was too brief. They desired more in-depth knowledge to be taught, enabling them to gain additional insights that could be applied throughout their volunteerism journey. However, they noted that the brief content might be a result of the short session duration. Therefore, they expressed a preference for longer sessions.

*I think it can go a bit deeper. It’s just that the depth can be, in my personal opinion, if time allowed, the depth can be more*

[K9, Female peer volunteer]

Furthermore, the older adults highlighted that the program venue was inconvenient. Some had to get up as early as 7 in the morning to arrive on time for the session. Despite the travel challenges, they expressed a desire for longer sessions to facilitate deeper learning.

*I find it’s really short because we [travel] from the east [and] we spend only two hours… the timing is not enough to share with us.*

[K04, Female Peer Volunteer]

Many older adults envisage more hands-on practice during the PFA program. Though older adults caught up during breaks and role-plays, having a specific time for small-group discussions regularly was highlighted by some older adults.

*I was thinking maybe it’s good to have maybe small group discussion during each session.*

[K11, Female peer volunteer]

A few older adults suggested incorporating various learning tools, such as case studies and better audio-visual materials for learning. Those older adults with less volunteer experience felt the need for more case studies to be discussed so that they are more prepared to handle the situations in real life.

*The other techniques like case study so examples of how we can approach the people… I never counsel anyone so I wouldn’t know, what [is] the real situation outside... I am bit worried to handle if someone suddenly cry…*

[K3, Female peer volunteer]

In addition, older adults highlighted several considerations that influenced their learning experience when engaging with older learners. They encountered issues with audio and visual clarity, such as some participants finding the projected fonts too small, requiring them to squint to read. Furthermore, the facilitators’ lack of use of a microphone led to poor hearing, making it difficult for a few participants to fully comprehend the content taught.

*As we age, our eyesight is not so good, we have to strain our eyes. I had to strain my eyes to read [the slides].*

[K1, Female peer volunteer]

*If you’re seated behind far away, you can’t hear much. I’m trying to figure out what he’s saying. So if you have a mic, then at least, you know, we can hear, everybody can hear.*

[K4, Female peer volunteer]

#### Future Learning Opportunities

Older adults highlighted the potential for future learning opportunities. Many expressed a desire for regular “top-up” sessions, either annually or semiannually, to revise the knowledge. Furthermore, they showed interest in gaining new knowledge through these regular sessions. A few older adults stressed the importance of regular sessions so that more peer volunteers from their respective organizations could participate and benefit from the PFA program.

*It will be good if there’s every six months or a year, we come back do skill upgrading.*

[K9, Male peer volunteer]

*I think we need to have more of this courses, for more people to be trained because now Singapore is aging.*

[K7, Male peer volunteer]

In addition, they valued the regular sessions as an opportunity to reconnect with their peers. They were eager to meet and share their recent volunteerism experiences, fostering mutual learning through casual conversations.

*A small group, then we will have regular meetups for sharing and to also impart certain message.*

[K11, Female peer volunteer]

## Discussion

### Principal Findings

This study explored the experiences of peer volunteers in the PFA program in Singapore, providing insights into their motivations, challenges, and the overall impact of their participation. All interviewed peer volunteers participated in all 4 sessions of the program. The sample was primarily composed of Malay participants (n=6), followed by Chinese participants (n=5), with one participant from each of the Indian and Javanese communities. While Singapore’s population is predominantly Chinese, Malay, and Indian, future studies could aim for a more balanced racial distribution to better reflect broader societal trends [39].

The older adults in this study cited various reasons for their continued involvement in volunteerism, including cultural values instilled by their families, peer influence, and the promotion of healthy aging. Through volunteerism, the older adults in this study were also able to actively engage with the community, fostering new friendships and a sense of purpose. This aligns with existing literature, with studies conducted in Japan, Australia, and the United Kingdom suggesting that the motivations for volunteerism in older adulthood were driven by community engagement, building new friendships, positive influences on personal well-being, and the desire to foster a sense of purpose [40-42]. Interestingly, altruistic attitudes, such as cultural values of graciousness instilled in them, were a key motivation for the older adults in our study. This contrasts with existing literature, where volunteers report motivations driven by egoistic reasons such as the development of a new skill and personal satisfaction [43,44]. This difference may be attributed to the strong Asian values that emphasize family cohesion and collective well-being [45]. As such, future studies can compare and explore volunteer motivations across different cultural contexts.

While the older adults in this study demonstrated a strong commitment to volunteerism, they also faced personal barriers, including difficulties in engaging with the community and physical limitations such as ongoing medical conditions or frailty. Chronic illnesses or frailty restrict their ability to participate in physically demanding tasks, limiting their ability to contribute as fully as they may have wished. This is consistent with studies conducted on older adults in Spain and Norway, which found that maintaining good health was a key factor enabling volunteerism as it was highly prioritized by older adults [46,47]. These challenges highlight the need for support systems that prioritize older adults’ health to ensure their ongoing participation in volunteer activities, ultimately improving both their physical well-being and their ability to contribute meaningfully to the community.

Cultural and social sensitivity emerged as another key theme in this study. Older adults encountered cultural taboos, particularly within the Muslim community, where there was a preference for interactions within one’s own ethnic or social circle. This limited broader community engagement, highlighting the importance of cultural sensitivity in volunteer programs. Additionally, gender dynamics were cited as a challenge, with female participants feeling more comfortable providing support to those of the same gender. These findings are consistent with research indicating that cultural and gender factors can influence interpersonal dynamics in volunteer settings [48]. This may be attributed to Singapore’s diverse ethnic composition, where cultural norms are critical. Furthermore, in the Asian context, where gender dynamics are often less openly discussed, it is possible that the older adults in this study feel more at ease interacting with individuals of the same gender [49]. This suggests the importance of addressing these sensitivities in designing inclusive volunteer programs that accommodate diverse cultural and gender preferences. Future research could explore how these factors shape volunteer experiences and inform the development of more culturally inclusive training programs.

Personal development emerged as another significant theme, with participants noting the value of peer interactions, role-plays, and experiential learning during the PFA program. These activities not only reinforced the content but also helped participants apply the concepts to real-world situations. This contrasts with a study of clinicians in the United States, where participants expressed a preference against interactive training methods [50]. The difference may be attributed to the older adult population’s preference for active, hands-on learning approaches, which has been noted in other studies [51]. Facilitators also played a crucial role, providing reassurance and fostering a supportive learning environment. This finding is consistent with research indicating that facilitators’ knowledge and support are integral to positive learning experiences [52,53]. Participants in this study also expressed a desire for more knowledge and confidence to handle real-life situations, aligning with findings from Japan and Haiti, where peer volunteer training boosted confidence in applying training strategies safely [54,55]. This highlights the importance of designing volunteer training programs that incorporate both theoretical knowledge and practical, experiential learning.

Designing inclusive training programs involves a holistic approach that considers accessibility, logistical support, practical learning experiences, and efficient course delivery, all of which contribute to enriched learning experiences and future improvement opportunities. Our study highlighted that the participants in our study desired greater comprehensive coverage of the content, with additional hands-on practice and small group discussions to enhance the training experience and add greater value to their volunteerism journey. Additionally, they expressed interest in incorporating other educational tools, such as case studies, to better assimilate real-world scenarios and mentally prepare themselves. This is consistent with studies conducted in Gaza, Korea, and Singapore, which emphasized that PFA trainers desire more in-depth training and new delivery approaches to psychologically equip themselves, enabling them to better support the recipients [25,52,56]. Additionally, older adults in our study emphasized the importance of regular sessions to stay informed about current information about PFA. Moreover, our study found that holding regular sessions would facilitate enrollment for prospective participants, providing them with comprehensive knowledge. These findings align with studies conducted in the United States and the United Kingdom, where participants envisage refresher sessions for PFA to further equip themselves with the latest knowledge [32,50]. Furthermore, several participants in our study, drawing on their prior volunteerism experience, expressed a desire for advanced volunteerism courses offering enhanced learning opportunities. This aligns with findings from studies conducted in the United States and Singapore, which highlight that individuals with volunteerism experience gain greater benefits from comprehensive training programs compared with basic ones [50,52]. Thus, a holistic approach to program design, which considers accessibility, practical learning opportunities, and continuous support, can enhance the overall success and impact of volunteer programs, and stakeholders can consider these recommendations in providing better support to the peer volunteers and, in turn, to the community-dwelling older adults.

### Limitations

This study has a few limitations. First, this study was conducted on English-speaking older adults and may not be transferable to non–English-speaking older adults in Singapore. Furthermore, Indians were underrepresented in this study. Hence, future studies can consider exploring the purposive sampling technique to study participants who are non–English-speaking and from diverse ethnic backgrounds to get a holistic understanding of the phenomenon of interest. Second, interviews were conducted with a convenience sample of older adults who volunteered to participate and hence could have harbored particularly positive views from those participants who were motivated to share about the PFA program. Third, a potential limitation is that participants were not explicitly asked to provide feedback on the interview guide during the pilot interviews, specifically on aspects such as question clarity, sequencing, and overall structure. Such feedback could have offered valuable insights for refining the guide and enhancing its suitability. This should be considered in future research.

### Implications for Future Research and Practice

Future research should explore the influence of cultural norms and gender dynamics in volunteer programs, particularly in diverse and multiethnic contexts like Singapore. Studies can investigate how cultural and gender considerations affect volunteer engagement and explore strategies for overcoming these barriers. Future studies should focus on the role of cultural sensitivity training and its impact on improving interactions among volunteers and community members. The physical limitations of older adults, including chronic illnesses or frailty, were identified as significant barriers to volunteerism. Future research should delve deeper into how health status influences volunteer participation and explore ways to accommodate volunteers with varying levels of physical ability. Moreover, longitudinal studies could assess the long-term health benefits of volunteerism and how these programs might mitigate the physical limitations of older adults. Stakeholders working with older adults, such as community partners and health care providers, could benefit from considering the cultural and age-related concerns highlighted in this study. Understanding the unique cultural values, gender dynamics, and health challenges faced by older adults is crucial for fostering more effective engagement and providing tailored support. This study highlighted the importance of hands-on learning, role-playing, and peer interactions in enhancing volunteers’ understanding and confidence. Future research should focus on evaluating the effectiveness of different interactive learning methods across various populations. Additionally, exploring the impact of peer-led sessions versus professional-led training could help refine approaches to fostering confidence and competency in volunteers. Research into the most effective formats for delivering such programs—whether through in-person, via web, or hybrid methods—could contribute valuable knowledge to improve volunteer retention and engagement. This study emphasizes that individuals with prior volunteerism experience benefit from a more comprehensive training curriculum. Hence, future research can explore the potential of offering both basic and advanced PFA programs for learners at different proficiency levels to enhance individual learning outcomes. While this study focused on short-term engagement with the PFA program, future research should explore the long-term effects of participation on older adults’ well-being, volunteer retention, and community impact.

### Conclusions

This study explored the experiences of older adult volunteers who participated in a PFA program in Singapore. Findings highlighted the complex interplay of cultural, social, and personal factors that influence the experiences of older adult volunteers. The findings suggest that training programs could be designed with cultural sensitivity, provide experiential learning opportunities, and support participants’ ongoing personal development to maximize their engagement and impact. Future research should further explore these findings to develop more inclusive, effective, and sustainable volunteer programs that empower older adults to contribute meaningfully to their communities.

## Supplementary material

10.2196/71810 Multimedia Appendix 1 Additional material.

## Abbreviations

COREQ: Consolidated Criteria for Reporting Qualitative Research
PFA: Psychological First Aid
