# Supplementary material for: Peer Volunteers’ Journeys Through Training and Engagement in Older Adult Communities: Descriptive Qualitative Study
Source: JMIR Aging. 2025 Jun 13;8:e71810. doi: 10.2196/71810 (PMC12207342; doi:10.2196/71810)
Supplement: Multimedia Appendix 1 [file aging-v8-e71810-s001.docx]

**Table S1:** COREQ (Consolidated criteria for Reporting Qualitative research) checklist

| **No.** | | **Item** | | **Description** | | **Section #** | |
| --- | --- | --- | --- | --- | --- | --- | --- |
| **Domain 1: Research team and reflexivity** | | | | | | | |
| Personal characteristics | | | | | | | |
| *1.* | | Interviewer/facilitator | | Which author/s conducted the interview or  focus group? | | Page 6 | |
| *2.* | | Credentials | | What were the researcher's credentials? *E.g.*  *PhD, MD* | | N/A | |
| *3.* | | Occupation | | What was their occupation at the time of the  study? | | Page 6 | |
| *4.* | | Gender | | Was the researcher male or female? | | Page 6 | |
| *5.* | | Experience and  training | | What experience or training did the researcher  have? | | Page 7 | |
| Relationship with participants | | | | | | | |
| *6.* | | Relationship  established | | Was a relationship established prior to study  commencement? | | Page 6 | |
| *7.* | | Participant knowledge of the interviewer | | What did the participants know about the researcher? *E.g. Personal goals, reasons for*  *doing the research* | | Page 6 | |
| *8.* | | Interviewer characteristics | | What characteristics were reported about the interviewer/facilitator? *E.g. Bias, assumptions,*  *reasons and interests in the research topic* | | Page 6 | |
| **Domain 2: Study design** | | | | | | | |
| Theoretical framework | | | | | | | |
| *9.* | | Methodological orientation and theory | | What methodological orientation was stated to underpin the study? *E.g. grounded theory, discourse analysis, ethnography,*  *phenomenology, content analysis* | | Page 5 | |
| Participant selection | | | | | | | |
| *10.* | | Sampling | | How were participants selected? *E.g. purposive,*  *convenience, consecutive, snowball* | | Page 5 | |
| *11.* | | Method of approach | | How were participants approached? *E.g. face-*  *to-face, telephone, mail, email* | | Page 6 | |
| *12.* | | Sample size | | How many participants were in the study? | | Page 7 | |
| *13.* | | Non-participation | | How many people refused to participate or  dropped out? What were the reasons for this? | | N/A | |
| Setting | | | | | | | |
| *14.* | | Setting of data  collection | | Where was the data collected? *E.g. home, clinic,*  *workplace* | | Page 7 | |
| *15.* | | Presence of non-  participants | | Was anyone else present besides the  participants and researchers? | | Page 6 | |
| *16.* | | Description of sample | | What are the important characteristics of the  sample? E.g. demographic data, date | | Page 6 | |
| Data collection | | | | | | | |
| *17.* | | Interview guide | | Were questions, prompts, guides provided by  the authors? Was it pilot tested? | | Page 6-7 | |
| *18.* | | Repeat interviews | | Were repeat interviews carried out? If yes, how  many? | | N/A | |
| *19.* | | Audio/visual recording | | Did the research use audio or visual recording  to collect the data? | | Page 7 | |
| *20.* | | Field notes | | Were field notes made during and/or after the  interview or focus group? | | Page 7 | |
| *21.* | | Duration | | What was the duration of the interviews or  focus group? | | Page 7 | |
| *22.* | | Data saturation | | Was data saturation discussed? | | Page 7 | |
| *23.* | | Transcripts returned | | Were transcripts returned to participants for  comment and/or correction? | | N/A | |
| **Domain 3: analysis and findings** | | | | | | | |
| Data analysis | | | | | | | |
| *24.* | | Number of data  coders | | How many data coders coded the data? | | Page 7 | |
| *25.* | | Description of the  coding tree | | Did authors provide a description of the coding  tree? | | N/A | |
| *26.* | | Derivation of themes | | Were themes identified in advance or derived  from the data? | | Page 7 | |
| *27.* | | Software | | What software, if applicable, was used to  manage the data? | | Page 7 | |
| *28.* | | Participant checking | | Did participants provide feedback on the  findings? | | N/A | |
| Reporting | | | | | | | |
| *29.* | | Quotations presented | | Were participant quotations presented to illustrate the themes / findings? Was each  quotation identified? *E.g. Participant number* | | Page 9-20 | |
| *30.* | | Data and findings  consistent | | Was there consistency between the data  presented and the findings? | | Page 9-20 | |
| *31.* | | Clarity of major  themes | | Were major themes clearly presented in the  findings? | | Page 9-20 | |
| *32.* | | Clarity of minor  themes | | Is there a description of diverse cases or  discussion of minor themes? | | N/A | |

**Table S2:** Interview Guide

| **Questions** | |
| --- | --- |
| **Motivation to be a peer volunteer** | 1. Do you have any experience with volunteering before? 2. Why did you sign up to be a peer volunteer?  - What motivated you to join the program?  1. Would you like to share any personal experiences or stories that inspired you to join this program? 2. How did you come to find out about the program? |
| **Training experience** | 1. How was your experience with the training program? 2. How well did you feel the training prepared you to be a peer volunteer?   Content of training   1. What were some topics covered in the training that you felt was most beneficial to you as a peer volunteer?  - Why are these topics important to you?  1. What were some topics covered in the training that you felt was not beneficial to you as a peer volunteer?  - Why did you think these topics are not as beneficial to you as a peer volunteer  1. Are there any other topics that you feel was not covered in the training but would be helpful to you as a peer volunteer?  - Probe further into why certain topics are important.   Delivery of training   1. What training methods were used in the program (e.g. didactic lectures, workshops, role-playing, online teaching, group work?) 2. What training methods did you find most helpful in helping you understand the content taught?  - Why was certain training methods helpful?  1. Do you think the training was adaptable to different learning styles and needs?  - If not, what changes would you recommend?  1. How did you find the training resources and materials?  - Were the training resources all sent online such as powerpoint slides? Or were there any physical guide book you received?  Were they useful? - Were there any training materials that you felt could have been improved?  1. How long was the training sessions in total? (Did it span through days?)  - Was the duration of the training adequate to cover the necessary skills? - If not, how long would you prefer it to be?  1. How did you find the pacing of the sessions?  - Was the trainer rushing to finish the content? - Were you given breaks in between?   Interactions among peer volunteers   1. Did the training program facilitate any interactions with other peer volunteers?  - Were there any group discussions/activities during the training?  1. Do you think it is important to interact with the other peer volunteers? 2. How could the training improve bonding amongst the peer volunteers?   Accessibility   1. How accessible was the training program in terms of location/ timing/ format? 2. Were there any barriers hindering you from fully participating in the training? |
| **Trainer’s feedback** | 1. How would you describe the competence of the trainers? 2. What were their teaching styles like? 3. What did you appreciate the most about the trainers? 4. How do you think the trainers can be improved? 5. What are some traits one should have to be a good trainer? |
| **Application of training in real-world situations** | 1. Can you recall the first time you applied the skills of Psychological First Aid that you learnt during the training?  - How was the experience? - How did you feel during and after the experience?      1. How frequently do you use the skills learnt in your daily life? 2. Can you provide some examples of situations where you applied the skills learnt during your training? |
| **Impact of peer support program** | Impact on beneficiaries   1. In your experiences and observations, how have the older adults that you have supported responded to your support?  - What were some positive outcomes that you witnessed? - Were there any unintended negative outcomes from your support? (If yes, how did you handle it?)  1. If you have not supported anyone yet,  - What are some positive outcomes that you think older adults will get from peer support? - Do you have any concerns regarding how older adults may respond to your support?   Impact on peer volunteers   1. How has being a peer volunteer impacted you personally? (Emotional, psychological, social)  - Could you give me an example?  1. Have you developed any new skills from this experience?  - Elaborate more about these skills and how you think will be helpful in volunteering.  1. Have you gained any new insights from this experience?  - How did this new insight value add to you as a person? |
| **Challenges encountered** | 1. What challenges did you face? (interpersonal, logistical, emotional) 2. How accessible are the volunteering centres for you? (Location, timing) 3. How did you overcome these challenges?  - Has the training program equipped you well with resources to handle these real-life challenges?  1. How is it like to volunteer as a male/female? 2. Do you feel comfortable to provide volunteering to females/male and likewise? |
| **Support and resources** | 1. We are approaching the last 2 questions for this interview. 2. What kind of support did you receive from the organisers running the peer support program?  - Was the support provided sufficient?  1. Any additional support or resources that will help you as a peer volunteer? |
| **Concluding remarks** | 1. Is there anything else you'd like to add or share about your experiences of the peer support program? 2. Do you have any advice or insights you'd like to offer any new peer support volunteers? |

**Table S3:** Themes, subthemes, codes and verbatim quotes

| Theme 1: Dimensions of volunteerism from motivations to resistance | |
| --- | --- |
| Subtheme 1: Intrinsic motivation to volunteer | Altruistic values (code)  Well, what I realize is I was too busy during my working days, young and teen days and I couldn't give much time to my parents, especially my mom. So when my mom passed away. [Started tearing] I realized that I should have given her more time, you see. So I just want to give back to the society to do good for others because one day I'm going to get old myself. So you know, it's better to do whatever I can do now, you know. (K01, Female Peer Volunteer)  I attended a course, a talk by Prof Kua la, that was in 2016, 2017, something like that ah. Uh, from there on, then I only realized that my mother is basically suffering from dementia, which I'm not aware of it. (K02, Male Peer Volunteer)  So, uh, at the same time, uh, sometimes I joke with people, oh, uh, it's to help myself to prevent having dementia because I'm learning something new. And, um, my father has mild, uh, dementia also, actually the, the, his the, the, his stage progress. So we can see that, that, uh, something's actually declining, yeah. So, so then I think to me is, is, is a very good initiative. (K03, Female Peer Volunteer)  Um, well, at that time I left my job for a while and then I have nothing to do so, and then with COVID, so, and then I joined the GRL at Tampines Changkat. Then, uh, the timing was just right. So they need, um, you know, volunteers to do the, uh, temporary relief fund. So I, I volunteered myself to help to fill out the forms and all that. And this is when I get to know, you know, uh, the residents and then know about their problems and then try to channel it to, you know, proper places. (K04, Female Peer Volunteer)  Well, uh, I see my father as a figure who do all this volunteer work in this life.… so it's already in my blood lah. (K05, Female Peer Volunteer)  It's just that when I first, when I resigned, so I'm just staying at home, then my friend Asiah, who the one who recommend me, asked me to join her to read stories to kindergarten school. So from there, after that, then uh she asked me to join, follow her to the AWE at the CC there. So from there, I like it. So until now, I've been continuing it. (K08, Female Peer Volunteer)  Well, I would like to do something that can be helpful to the community. Right. So basically you know, because before that I'm also doing, you know, all this of services already for the seniors. (K10, Male Peer Volunteer)  Basically the interest for signing up is just like, uh, if for me is, my motivation is I know some about, um, some facts, uh, true facts about, uh, uh, of, in helping and or counselling a person. It, uh, it would be, uh, helpful for me, uh, in my, um, with my tutees. (K12, Female Peer Volunteer)  I find that, uh, uh, to help people because I had gone through the, uh, the stage you see at one time, a period of time you see, and then from that time, it makes me that I want to touch more and especially on those like, those single moms, you see. (K13, Female Peer Volunteer) |
| Subtheme 2: Personal hurdles in their volunteer path | Resistance in engagement (code)  Especially with the yellow ribbon. You know, when you attend to the, to some cases where the, the, the children is uh, inside and then you have to take care of the parent, the parents, and then the siblings. Some of them, they're very uh, what should I say? Uh, they, especially like, it's the inmate going in and out, so the family like can't be bothered already with them. So how are you going to ask them to visit the, the child, the the son or the, the daughter in prison. Then they, you know, I really encourage them, say, you know, please don't give up hope. Say ah, this son hopeless already, you know, so many times in and out. How are you going to give them the assurance that the son or the daughter will change? (K04, Female Peer Volunteer)  They just, they just doesn't want to talk to us. Like if we go to their house, they don't want to talk, then what can we, what can we say, right. (K06, Female Peer Volunteer)  I think I've got err what is that.. aphasia where you, you know, sometimes my train of thoughts go out, run out of it. Sometimes when people talk to me, I can't interpret properly. (K10, Male Peer Volunteer)  I think it's the resistance of the, the, the people like when we try to talk or whatever, there is certain resistance or there is also, there is also fearful or we might, we might say the wrong thing to agitate people especially the senior, yeah. (K11, Female Peer Volunteer)  Awareness of one’s physical capabilities (code)  Because I am, uh, quite small build, I don't have much strength. So things are very physical. I think probably I, I would not be able to help because sometimes some, depending on some situation, let's say they're doing exercise or they're walking, uh, for those who are weak, we may need to provide them physical support, like to support them so that they don't fall down. (K03, Female Peer Volunteer) |
| Subtheme 3: Cultural Boundaries and Social Sensitivity | Communication among gender dynamics (code)  Not easy to understand a male, what he's thinking. A female, when you look into her eyes, you talk to her, you can understand more. You can know what she's going through, what she's feeling, what she wants to say. But she's not able to say for a female it's much easier because you dare to look into her eyes for a male and you look in your eyes also don't want to get wrong ideas. (K01, Female Peer Volunteer)  Female and female. It's just easy to talk. I think it's just the talking. It's about the talking that would affect the person's heart. So let it be free of mind and that is where to choose the female and female is better rather than to create any problem after that. (K05, Female Peer Volunteer)  We have to agree that we must have another person with us. I cannot just be alone to, to meet him, you see. Okay. I must have another person to, to be around. It's not one to one, uh, if that, if that's a male. (K06, Female Peer Volunteer)  Because for female and male, there is a gender difference that we need to be more mindful, like for female, we can touch the female, can consult and all this, but for male all this, we need to be more careful, more mindful on our gesture and all this. (K11, Female Peer Volunteer)  It's just, like, even among our own, um, volunteers, if you are closer to some, uh, the other gender's friend, then the person's wife may not be happy seeing that you are so close with her husband. Then you will see, uh, some negative, uh, uh, reactions from the wife. (K12, Female Peer Volunteer)  You see, for us in the in the so-called religious or Muslim way, we cannot interact too much with the opposite opposite side. This is one of the challenge. (K07, Male Peer Volunteer)  Cultural boundaries (code)  Initially in getting participants, you know, Malay always thinking about what taboo things that should not be said openly. But when I talk to them, when I explain to them, then only now, basically these people, they able to accept us. (K02, Male Peer Volunteer)  Especially, as I say, it's on our side, like for the Malays, uh, elderly, right. To like overcome that, how we wants to get them, like, how are we going to approach them that to ask them that, uh, we can really give them a help, you see. That's, that's our challenge now. (K13, Female Peer Volunteer)  You see at this center is a great challenge for me in terms that the medium of communication is in Mandarin and my Mandarin sucks. (K10, Male Peer Volunteer) |
| Theme 2: Empowerment through collaborative learning | |
| Subtheme 1: Personal development | Enhanced personal growth (code)  So it was nice la it was very knowledgeable. I must say we learn a lot and what we knew about like meditation from Mrs Wee, it became more and more for us to confirm. (K01, Female Peer Volunteer)  The PFA workshop where it really broadened our mind and then we learn more about things which is, uh, we could say is, uh, under this kind of scientific kind of, uh, wordings that come out with it. (K02, Male Peer Volunteer)  One of the session there's grief, talking about grief. So, so then I think that mindfulness training will make us aware how we will feel because there's some mind body connection. So, so then it's, it's supposed to be, I think it is a whole package. It's a very unique package, which, which after attending so many, uh, I mean, others counseling training, I never got to experience it. So this one I find very unique. (K03, Female Peer Volunteer)  Wow, uh, I feel great I have, uh, new knowledge. (K05, Female Peer Volunteer)  For myself, for myself, it's positiveness, it's, it's, it's, uh, positiveness lah rather than negative lah…. . For me, I try myself to be positive to to accept whatever is being, you know, in life. (K06, Female Peer Volunteer)  I think should be uh don't change it to become too personal, wait will make you feel down. This is what I see. Don't get too personal. Yes, personal can be, but there's a way to resolve. Don't keep to yourself. You will. You will end up being depressed. (K07, Male Peer Volunteer)  I think it's really inspired me because when I listen to whether Professor Kua or Mrs Wee or Prof Shefaly talk, it's really inspired me to know how to approach all these people. Because usually when, uh, it's just that our, um, what I do is simply just at that time, I just do whatever I know. So from these courses. I think I learned a lot, uh, how to approach, how to handle, how to talk, so all these things. (K08, Female Peer Volunteer)  It's a continuation for me to upgrade myself. And also, because through the, through my work, I know I come across a lot of stressful people, uh, but I'm not, I'm not professional enough to see whether they are really regular, normal stress, ordinary stress, or is it come to a point whereby there's a, uh, some so called, uh, depression or mild depression type, right. So I thought this, this, uh, workshop will help me to, to, uh, be a bit more, uh, sensitive in picking up some of these signs and, and, uh, telltale signs, right. (K09, Male Peer Volunteer)  Oh, it's an eye opener to me. I love it so much, you see, when, uh, when the, uh, Professor Kua talks and then when, uh, Professor Shefaly talks, you see, then I was, I was sitting and then I was saying that, uh, this is the journey that I had, I had gone through, but I don't know that, you know, we can go even deeper. (K13, Female Peer Volunteer) |
| Subtheme 2: Mutual learning and through peer interactions | Learning from peers (code)  You get different ideas from them. You learn more things from them, they learn from you. It's good to exchange ideas. (K01, Female Peer Volunteer)  In a way, it will be good because, uh, yeah, actually, I remember one of the session that the person next to me, she wanted to chit chat and ask something, but, but because of, I think, Prof is continuing with his teaching, because, uh, the other group is mainly, uh, because the other group are Malays ah. So, so then cultural wise, it's quite different… to, I think to understand each other's cultural difference (K03, Female Peer Volunteer)  Yes, of course we have to, of course, that's one of the way to learn. And, you know, from each other, of course we, we are from different, different section, right. We are from different sector. (K06, Female Peer Volunteer)  Yes, yes, we when break time, we mingle around, we talk about. So we ask question, hey, I heard about this, this this so they share share with us from their own personal life, personal sharing and so then we get become more closer with other participants. (K07, Male Peer Volunteer)  I think yah, it's important because when we interact, at least we know something, uh, what's come to their mind. At least we gain something from them also. So rather than we ourselves not interfering with them, not mixed with them. So it's just that we know our own thing, but we did not know what other people think. So when we interact, like that day, I talked to some of the Kwan Ming San people. So I know what they are doing. I know how they handle all these people. So all this gains me more experience to talk to them. (K08, Female Peer Volunteer)  It's helpful for, for, for understanding of everyone experience and how people are doing and coaching and all this. So, yeah, I think it's, it's, it's, it's important for, for, for interaction. (K11, Female Peer Volunteer)  Because, uh, you, each of us have our own experience. So, uh, there may be certain cases, uh, that they can share, I mean, case study, they can share it. Definitely you will benefit from it. I personally, I've used it. (K12, Female Peer Volunteer)  Actually, to me, it's best to have more interact because in that class, we don't know each other. Uh, so if he given us more, that mean the bonding is is much more, uh, strong, you see. So that we can share more on, uh, our experience to what they are, what they experienced too. So that we can become more, uh, confidence of, on our volunteers, you see, as a volunteer. (K13, Female Peer Volunteer) |
| Subtheme 3: Impactful Teaching and Facilitation | Qualities of trainer (code)   - How trainers enhances their learning experience   They were too good too good especially Shefaly. She was so lively and bubbly that I wanted to see more and more and more of her. (K01, Female Peer Volunteer)  The three trainers, they are professional. They are really professional and doing all this ah is supposed to be their, their their blood in it, I say, and then because of the passion that they have ah, so that is where they never really rested one thing. They keep on giving us, uh, knowledge after knowledge. (K02, Male Peer Volunteer)  I think I learned from each one of them. Like, like when Prof Kua uh, talks, I feel that he, he, he made me feel he's very warm. And then uh, Dr Shefaly, uh, she's very good in, uh, the way that she carry herself. I think that can be her style lah. But, but, but, but yeah, it's, I, I learn from it. And then, uh, Mrs Wee because she, she's a mindfulness teacher and I'm learning to, to teach mindfulness. So of course I, I also want to learn from her. (K03, Female Peer Volunteer)  I find they are quite dedicated with their, their work. Uh, the enthusiast is there, you know, they, they, they share, you know, with us. Yeah, I find that they're very good. (K04, Female Peer Volunteer)  Professor Shefaly, she is a person who is, she has a package. Yeah, she can act. The way she talked to us, with her action, everybody just stood and stare and we, all of us are immersed in the way she talked. (K05, Female Peer Volunteer)  Their sharing, their sharing their knowledge and their especially their determination. And they have a great what do you call it, they always have a big ears to listen to us. (K07, Male Peer Volunteer)  I appreciate how they want to teach us, how they want to help us to teach other people or helping other people which they also did not know who. Only for us, we know who we want to help. So for them, they teach us really from their heart, what I know is that. They really help us to tackle the problem, to know how to deal with the problem. So, uh, I think they are very good the way they teach, the way they present, and how they teach us so that we really knows how to deal with all the problem, yeah. For me, they are very good. (K08, Female Peer Volunteer)  If you ask about experience, I really love it. I, I see how Prof Shefaly talk ah really inspired me. uh, that's what I think I will teach my old people at the CC and how Prof Shefaly talk ah, I think I will follow her step, talk to them, yeah, correct. It's good la for me. (K08, Female Peer Volunteer)  They... think the way they present and they deliver the topic well, yeah and they are very knowledgeable on the topic and they can put it in a very, how to say, easy to to, to understand in a way, yeah, very lively. (K11, Female Peer Volunteer)  Um, in fact, uh, like, um, is, uh, like Professor Kua okay, when he talks about the grief, uh, that how I overcome it and how to overcome it, okay, I, I really like the way, and then from these, uh, Professor Shefaly, when she says that how to, how to overcome on the, uh, people who are in, in the level of education, the girl is so high up, but, and so surprising that she also have the depressions. So, it really touched me. So that's, uh, really good la the way they, uh, they talk to us, they give us an opener, how to overcome in futures, you see. (K13, Female Peer Volunteer |
| Subtheme 4: Experiential and Applied Learning | Methods which enhance their learning (code)  Especially the role play it showed you like reality what could happen or what will happen or what can happen, you know. So that was really like an experience thing. Very good because learning from slides and all is quite normal, you read. But when you have a role play, you really understand it more than you sometimes put yourself into that shoes, you know, and see, ok, how it is like. It it was, that was really very good. (K01, Female Peer Volunteer)  Uh, to help us I think to see, uh, his way of roleplay is, is he got someone to play as, uh, uh, in distress and then the other person tried to talk to it. So, so I think in a way that within a short time we can identify what are things that maybe will work. What will not may not work rather than covering the theory, like, you know, how you should sit because the, the, the usual counseling one that I attend is they will say, Oh, you need to sit, what, open posture, blah, blah, blah, blah, blah. Then it's quite dry la. So I think he do two in one, uh, because in the role play, let's say if the person didn't sit properly, then you will see, Oh, what is the consequence? Like, the person end up touching too much, then we'll see, oh, yeah, what is the consequence? So it's like two in one. (K03, Female Peer Volunteer)  Cause role play was uh, few of them during the role play, they they act as the you know, um, their experience with with, uh, their, you know, whoever that they they, they met la, you know, so in a way they, they share with us what happened and all that. Then from there, we discuss, you know, what is right, what is wrong, the approach and then, you know, your tone of voice, you know, yeah. So from there, you know, we learn how to tackle the situation when you're with somebody, your body language, you know, so all it's all in in a way (K04, Female Peer Volunteer)  Uh, they asked us experience our experiences, uh, that we had that is similar to the topic. So the experiences, or rather the sharing also benefit us who listen. To me, it is an example which can be stored in our mind. So, you know, different people have been those kind of different experiences. we learn from that and like what I said it's taught in our mind so um it gives it benefit us. (K05, Female Peer Volunteer)  Mostly their experience lah. The person experience of certain, certain, uh, engagement lah. Then we, we, we learn from there lah. Then we can know how to overcome that situation regarding about this, maybe, uh, if I face, uh, uh, situation like that, then we can, we can prepare ourselves also la. This is one of the way that means we are prepared to answer whatever their questions, that means if they any problem or whatever it is, then we can, we can, uh, relate. (K06, Female Peer Volunteer)  Helpful to me is the training of like, um, how they act, the way they act, uh, is like, uh, you, how to say, is just like you have more confident when you see the act. So you know, oh, this is how you need to tackle the problem. This is how you need to talk to the people, how to approach them. Yeah, from that, uh, scene, I think that one is more better rather than just talk, all this thing, yeah. (K08, Female Peer Volunteer)  Prof Kua is good, he know how to really yeah, based on his experience and all this, he, he is, he's very good. He, the delivery, the, the, the delivery of the topic and, uh, how he gathered people to do the, the role play and all this, I think he's very pro (K11, Female Peer Volunteer)  Um, you you can you observe how, or say, um, certain, um, things were being presented, uh, to right in front of you, and then you have a better, how to say... You see, right then you better, uh, you understand better in that sense. And then to me, um, I'm a visual person. So, um, uh, it will have a deep impression in me ah and then, uh, through the role play you can see, uh, how other people reacted. (K12, Female Peer Volunteer)  Uh, I mean, for, for us, like the old age ah, we prefer like a role play. So because when there is a role play meanings like action is there then ready really on the, on the theory. It, we, we mix make us like, you know, like very dull, you see. (K13, Female Peer Volunteer) |
| Theme 3: Recommendations for designing inclusive, holistic training programmes | |
| Subtheme 1: Depth and Quality of Learning | Preference for in-depth knowledge, longer sessions and accessible location (code)  It was, you know, out of the way, difficult and all the first time when we went, lost the way also couldn't find it and 2nd we came to know the second time we you know landmarks ah we tell the driver, these are the landmarks and all the google. Google sometimes shows them a longer route, you know not not the road and then we are late. (K01, Female Peer Volunteer)  Only thing. Yeah, too early it started. Because we stay far away, we have to leave early. You know, we have to leave early and get up early. So we're, like, rushing a bit (K01, Female Peer Volunteer)  I find it's really short, too short, that's why I say we go all the way because we are from the east. We go all the way there in the morning. Then we spend only two hours. Yeah, uh huh. I mean like uh, the only thing I say is not um, the timing is not enough. Two hours is, I find it's too short for them to yeah, to share with us. (K04, Female Peer Volunteer)  I should say not really, but it, uh, how to say? That's the beginning of the lesson. Um, it's still come back to you, how you apply. It's very basic. (K05, Female Peer Volunteer)  I think will be we have to learn more ah. It's not enough, uh, just to, you know, engagement is there. There's an engagement to, to create awareness. Uh, I think go to a deeper, deeper perspective. I think we have to, we have to have a more, more, uh, like, you know, we, we just, we just had that around four times. That means, uh, within one month, you see. Of course, it's not sufficient. (K06, Female Peer Volunteer)  Location wise is, uh, of course for me, uh, if you, if you ask me, I also don't know how to go. Lucky my partner, uh, she's from Punggol also, she knew where is the place. So we try our best to drop at Harbourfront. (K06, Female Peer Volunteer)  Only the the the the problem actually the distance. I'm staying in the east where I need to go to Alexandra Hospital. If you take a train also it takes you about 1 and a 1/2 hours. So is the distance. (K07, Male Peer Volunteer)  So, uh, in fact, I, I think it can, it can go a bit deeper. Uh, It's just that the depth can be, in my personal opinion, if time allowed, the depth can be more so that, so that we can, we can, uh, hone our skills a little bit more. (K09, Female Peer Volunteer)  With the during the four sessions or so, some of these are, I think, crazy, I would say so basic kind of things. (K10, Female Peer Volunteer)  Far for me because I'm staying at east site, so it's far for me. Uh, so I need to take a train and change the bus. Then it will take about one hour to reach. I don't drive and all this, so I take public transport. So it's like an hour or so to for public transport, so it's considered far for me. And the place itself is not so easily accessible. (K11, Female Peer Volunteer)  I think the duration is short then, uh, quite, um, the content itself is quite short, in the sense that, uh, the training itself a little bit short, and the content not so much. There's not much practice, even though we have a few, uh, uh, role play, but then, uh, the delivery of the contents, the topic and all this maybe can further enhance. Yeah, quite, quite, quite like touch and go, quite not in depth. (K11, Female Peer Volunteer)  Because she actually wanted us to have some, something like a roleplay or something like that. Something that she wanted to do la but right now I cannot remember. Ya, then she said something that she wanted to elaborate but due to the time constraint so cut short. (K12, Female Peer Volunteer)  Because, uh, it's actually two hours is not enough. Because the time is only like two hours. So maybe give us like, uh, more than two hours. (K13, Female Peer Volunteer)  Course delivery and structure (code)  So maybe in case, you know, in future you, you have more courses coming up, then maybe you can add another hour or two, you know, per course yeah. (K04, Female Peer Volunteer)  I think so. Uh, yeah, we, we, we, it'll be good. Let's, that's why I say because in future, if there's a future class, uh, it'll be good to say to to, to a small group, we can break into a smaller group, right. Four or five, or three or four person, uh, together. (K09, Male Peer Volunteer)  Yeah, if you think of activities, yeah, small group discussion maybe that will help. (K12, Female Peer Volunteer)  If I want to consider adding roleplay, one or two more roleplay, have small group discussion, that would be about 10 hours. (K12, Female Peer Volunteer)  I prefer to have like more than two hours, like at least three hours. It won't be rush, you see. (K13, Female Peer Volunteer)  Preference for more hands-on learning (code)  I thought if we, to let us have more practice, then no lah. Because the roleplay is actually a bit like a demo for us. We didn't get to try it out. (K03, Female Peer Volunteer)  So you know, I don't know what the others have gone through so it's good that uh, no doubt we do have some uh what the play ah, uh, role play yeah. So if you can have more of that and then sharing, uh, sharing, sharing, uh, you know, your your, uh, experience and all that. I mean it will help, you know. (K04, Female Peer Volunteer)  So more to hands on is much better because to for us senior or in our group is hands on is much better than reading. (K07, Male Peer Volunteer)  I think that will be very helpful to say, okay, this could be a kind of situation or this could become situation how how this person handle well and not well in a more in depth, uh, analysis that we can have a more in depth discussion in this in this particular past, uh, uh, program (K09, Male Peer Volunteer)  What I envisage is that there's more hands on practice for the whoever is going through this course. (K10, Male Peer Volunteer)  Yeah, I was thinking maybe it's good to have maybe small group discussion then, uh, like, uh, small group discussion then roleplay certain, uh, yeah. Then, uh, there's more, I think, maybe there's more small group discussion or small group support that maybe we can slowly build up from our first lesson until the end. (K11, Female Peer Volunteer)  But personally, I would prefer some more hands on, yeah. Maybe not in front of the whole class, but, uh, small groups within the peers ourselves to break into small little groups if there are more time. (K12, Female Peer Volunteer)  The need for applied examples (code)  You know, uh, then I was thinking about the video. I mean, the real case or in and all, some, uh, so called, uh, real site visit, you know, maybe one of the days in the future, go to real site visit to say, okay, how, how you guys are handling this, you know, and, and, and then, and then one more thing is talk about, uh, learning from, from, uh, uh, the real person, people like you, who are in this profession, how you handle your own stress when you're seeing all this, right. (K09, Male Peer Volunteer)  The other one is like maybe case study so that examples of, of how we can approach. Sometimes just examples of like, uh, for example, the, the, the thing that she mentioned was like a student, uh, having difficulty not coming out because these are real cases. If, if, uh, for us, if we just learn. But even I am trained in counselling, right? After that, I never counsel anyone. So I wouldn't know, uh, what are the real situation outside. (K03, Female Peer Volunteer)  Maybe a practice. But that practice, uh, would be the real person come to us. (K05, Female Peer Volunteer)  Probably if they invite us to go to the IMH, that could, that will open our, uh, how to say experience, give us experience that, oh, depression is not a simple thing. From depression then it, it, it become, how to say more… If it's out of control, it'll become more worse. (K05, Female Peer Volunteer)  And then we can have a group discussion to say, okay, this particular video, I mean, this particular real setting, uh, what can we learn from it or what can we, uh, what, what can we do better and things like that. So that I think will help us to prepare ourselves in a more, uh, uh, real, real environment. (K09, Male Peer Volunteer)  More on practice on the tool. The, the skill or, or, uh, the what? The theory is theory. But there is a delivery where we need to use the skill and the skillset and all this, which we don't have really much time for us to really, uh, practice on it or, or something like that la. (K11, Female Peer Volunteer)  Audio and visual clarity. (code)  So, you know, sometimes if you, if you're seated, you know, behind, far away, you can't hear much. I'm trying to figure out what he's, what he's saying. So if you have a mic, then at least, you know, we can hear, everybody can hear. (K04, Female Peer Volunteer)  For us, this, uh, uh, this age group, uh, because, uh, we all have the, um, 'laohua' (presbyopia). So we all feel that it's too small for us to read. (K12, Female Peer Volunteer)  At that time, first time when they give us the so-called something like a note but it is too small, too small for we got difficulties in reading. (K07, Male Peer Volunteer)  Ohh yes, yes, yes, there was some the the the font was very small, so it's supposed to be larger, but they couldn't help it, you know. See as we age, our eyesight is so not so good, you know, I mean, we can see, but we have to strain our eyes like I had to strain my eyes to read that you see. (K01, Female Peer Volunteer) |
| Subtheme 2: Future Learning Opportunities | Frequent sessions to enhance knowledge and peer interactions (code)  As for me to, to just to add on or whatever, I think it is best to bring it more often, know more often so that we I can send another set of my trainers to go there and try to pick up as much, uh, experience and much, uh, the same from them, knowledge from them. (K02, Male Peer Volunteer)  Ok, uh, actually what I think we need to have more this courses for more people to to be to be trained because now Singapore is aging. (K07, Male Peer Volunteer)  But, but the thing is that it will be good if there's some maybe every other six months or a year, or maybe at least a year, we can, we can sit up, sit, come back to, to hear any more news, you know, any more, uh, skill upgrading, I mean, from, from, from Mind Science Centre. I think that would be good. So for the, for the volunteers to. To learn new things, right. (K09, Male Peer Volunteer)  That's mean we need to have a time which can we call them, talk to them, at least we make, uh, around two months, one time, make call with all the volunteers, give them a talk or what, talk to them, ask their, uh, how's the, how's the life is, all these things. So I think it's more better to like more interact with them because now after these courses. (K08, Female Peer Volunteer)  Well, if you say have a regular meeting or gathering or things like that. I think that would be good, right. (K10, Male Peer Volunteer)  If let's say a small group, uh, uh, a group of those, uh, you know, maybe the support group, then we will have maybe regular, regular meetups for sharing, for caring and all this. And then to, to also impart certain, uh, message and all this, then there's a small group and all this will come together. (K11, Female Peer Volunteer)  I prefer like, because right now, some of them from the West, and I'm from the East. So it's best that if we can, you know, once in a while, we can have the, uh, the meetup again with the same class so that we can always share our experience, our knowledge, you see. (K13, Female Peer Volunteer) |
